# Radiographic features predictive of recurrence and survival after surgical resection of perihilar cholangiocarcinoma

**DOI:** 10.1016/j.heliyon.2024.e28805

**Published:** 2024-03-29

**Authors:** Julaluck Promsorn, Panjaporn Naknan, Aumkhae Sookprasert, Kosin Wirasorn, Jarin Chindaprasirt, Attapol Titapun, Piyapharom Intarawichian, Mukesh Harisinghani

**Affiliations:** a Department of Radiology, Faculty of Medicine, Khon Kaen University, Khon Kaen, 40002, Thailand; b Department of Medicine, Faculty of Medicine, Khon Kaen University, Khon Kaen, 40002, Thailand; c Department of Surgery, Faculty of Medicine, Khon Kaen University, Khon Kaen, 40002, Thailand; d Department of Pathology, Faculty of Medicine, Khon Kaen University, Khon Kaen, 40002, Thailand; e Department of Radiology, Massachusetts General Hospital, Harvard Medical School, 55 Fruit Street, Boston, MA, 02114, USA

**Keywords:** Perihilar cholangiocarcinoma (PCCA), Surgery, Recurrence, Prognostic factors, Recurrence patterns, Recurrence-free survival, Overall survival, Biliary tumor

## Abstract

**Objective:**

To study which radiographic features were associated with recurrence and adverse outcome in patients undergoing surgical resection of perihilar cholangiocarcinoma (PCCA), as well as to evaluate the imaging patterns that signify recurrence after the resection of PCCA.

**Materials and methods:**

This study was conducted in a solitary tertiary center and utilized a retrospective, analytical, case-control design. The study population consisted of patients with pathologically confirmed PCCA who underwent surgical resection and were subsequently followed up from January 2009 to December 2017. A total of 77 patients were enrolled in the study and were categorized into two distinct groups, namely recurrent and non-recurrent. The analysis encompassed the examination of demographic data and recurrence patterns. Additionally, survival and multivariate analyses were employed to assess radiographic imaging data and surgical information.

**Results:**

Seventy-seven patients diagnosed with PCCA based on pathological evidence were included in the study. Among the participants, there were 28 females and 49 males, with ages ranging from 41 to 81 years (mean age of 60.65 ± 7.66). A noteworthy finding was the recurrence rate of 65 % observed following surgical resection. The presence of regional lymph node (LN) metastasis, adjacent organ invasion, and surgical margin emerged as the three independent factors that exhibited a significant association with recurrence after post-operative resection (p = 0.023, p = 0.028, and p = 0.010, respectively). The patients with PCCA who experienced regional LN metastasis had a median overall survival (OS) of 22 months, which was significantly lower than the 46 months observed in those without regional LN metastasis (p < 0.018). Furthermore, the individuals with regional LN metastasis had a death rate that was 2.08 times higher than those without (p = 0.040). In addition, those with adjacent organ invasion had an OS duration of 21 months compared with 52 months in those without (p = 0.008), and the rate of death was 2.39 times higher (p = 0.018). Patients with an R1 resection margin had an OS duration of 36 months compared with 51.56 months in those with an R0 resection margin (p = 0.006), as well as a 2.13 times higher rate of recurrence (p = 0.010) and a 2.43 times higher mortality rate (p = 0.013).

**Conclusion:**

The presence of regional LN metastasis, invasion of adjacent organs, and R1 resection margin were identified as distinct factors that are linked to both disease recurrence and reduced OS. Local recurrence, as well as the spread of cancer to distant organs such as the lungs and liver, were frequently observed patterns of recurrence. To enhance the precision of staging, prognosis, and treatment, the inclusion of periductal fat or invasion of adjacent organs should be considered in the staging system for PCCA.

## 1 Introduction

Cholangiocarcinoma, a biliary tumor of malignancy, exhibits a notably elevated frequency in the northeastern region of Thailand due to the prevailing presence of chronic opisthorchiasis [[1], [2], [3], [4], [5], [6], [7], [8], [9]]. The incidence rates for males and females are reported as 44.3 and 17.6 per 100,000, respectively [10]. The classification system employed by the American Joint Commission on Cancer (AJCC) categorizes cholangiocarcinoma into three distinct subtypes, namely intrahepatic duct, perihilar, and distal [[11], [12], [13]]. The perihilar subtype, referred to as perihilar cholangiocarcinoma (PCCA), holds the distinction of being the most prevalent, accounting for a substantial 50–80 % of all cases [[14], [15], [16], [17], [18], [19], [20], [21]], followed by intrahepatic and distal CCA.

PCCA exhibits an aggressive malignancy, as evidenced by the fact that less than half of the cases are resectable at the time of diagnosis [22,23]. Moreover, even among patients who are suitable for resection, the 5-year overall survival (OS) rate following surgery with curative intent ranges from a mere 20 %–42 % [24]. The treatment of PCCA necessitates a major hepatectomy with bile duct resection, caudate lobectomy, and regional lymphadenectomy in order to achieve a tumor-free resection margin and optimize survival outcomes [[25], [26], [27]]. Despite improvements in the outcomes of curative resection for PCCA, the 5-year survival rate remains disappointingly low.

The recurrence rate of PCCA after curative resection during the follow-up period ranges from 50 % to 76 %. Predictors that are associated with worse survival outcomes in terms of recurrence include the status of the resection margin, tumor differentiation, vascular involvement, presence of lobar atrophy, LN metastasis, tumor size, poor performance status, and elevated CA19-9 [[28], [29], [30], [31], [32], [33], [34], [35], [36], [37], [38], [39],^39^]. Several factors have been identified that are associated with more favorable survival outcomes in PCCA. These factors include curative resection with a cancer-free margin, well differentiated or papillary adenocarcinoma, and absence of LN involvement [29]. The patterns of recurrence after surgical resection can be categorized into two types. The first type is distant metastasis, which occurs in 40 % of cases and includes distant LN metastasis, intrahepatic metastasis, peritoneum metastasis, lung metastasis [30]. A prior investigation on the survival results of curative resection for PCCA in the northeastern region of Thailand discovered that patients lacking LN metastasis exhibited superior outcomes [40]. Adjuvant chemotherapy, such as gemcitabine and cisplatin, has been employed as a systemic treatment modality subsequent to the surgical resection of PCCA [[41], [42], [43], [44]]. Recently, the Food and Drug Administration (FDA) of the United States (US) has sanctioned pemigatinib as a targeted therapy for CCA, which exerts its effects directly on the fibroblast growth factor receptor (FGFR) gene and has the potential to extend the median progression-free survival of patients [45]. Additionally, immune checkpoint inhibitors like durvalumab have been utilized in clinical trials for the management of biliary tree tumors [46]. Permitting the treatment of resectable intrahepatic cholangiocarcinoma, other locoregional treatments, such as percutaneous radiofrequency ablation, have shown to be associated with fewer complications post-procedure. Moreover, they have the potential to extend the period of local tumor progression-free survival [47]. It is crucial to conduct further investigations, both in vitro and in vivo, particularly utilizing animal models in collaboration with scientific societies dedicated to cholangiocarcinoma (CCA). These investigations would contribute significantly to enhancing our understanding of the intricate nature of premalignant lesions and the progression of CCA from an early stage to an advanced stage [48].

Previous studies have examined prognostic factors related to survival outcomes and recurrence-free survival in PCCA; however, the results have been inconsistent. The radiographic prognostic factors associated with recurrence have not been thoroughly described or understood. Consequently, analyzing the radiographic features associated with recurrence may aid in treatment planning and prognostic outcome estimation. Additionally, there has been no investigation into the imaging features associated with post curative resection of PCCA in northeast Thailand, which has the highest incidence rate in the country. Therefore, the objective of this study was to identify the pre-operative radiographic features that are linked to post-surgical resection recurrence and to assess the imaging patterns that indicate recurrence following resection of PCCA.

## 2 Materials and methods

### 2.1 Patients

This retrospective analytical study was granted approval by our institutional review board, and the necessity for patient consent was waived. Throughout the period spanning from January 1, 2009, to December 31, 2017, a total of 169 patients with pathologically verified PCCA were identified. Among these individuals, we included those who underwent surgical resection, possessed pre-operative CT or MRI accessible on PACS, and possessed validated post-operative follow-up data. We eliminated individuals who 1) had PCCA confirmed through pathological examination but did not undergo a surgical procedure, 2) received surgical treatment but were unable to be contacted for further monitoring, or 3) underwent surgical resection but had missing preoperative imaging findings. Based on these specific criteria, a total of 92 patients were excluded from the study. Among them, 52 individuals were unable to undergo curative resection due to the advanced stage of their disease at the time of diagnosis. Additionally, 31 patients could not be included in the analysis as they did not have post-operative imaging and were considered lost to follow-up. Furthermore, 9 patients were also excluded due to the unavailability of preoperative imaging results on PACS. After excluding these cases, the remaining group consisted of 77 patients who were further divided into two subgroups based on their outcomes. One subgroup included patients who experienced recurrence after curative resection, while the other subgroup comprised patients who did not experience recurrence.

### 2.2 Imaging analysis and data collection

All patients' epidemiological data and pathological reports were obtained from the hospital's electronic health object (HO).

A radiologist with over 10 years of experience in abdominal imaging and an early career radiologist with over 2 years of abdominal imaging experience evaluated the pre-operative abdominal CT or MRI using a 2000 × 2000 PACS workstation. Scans closest to the surgery date were analyzed at random with the researchers blinded to all clinical information, and results were determined by consensus. Imaging features examined included tumor staging according to Bismuth-Corlette classification, tumor type (periductal, mass forming, intraductal, combined), underlying chronic liver disease, tumor size, lobar atrophy, macrovascular involvement, regional lymph node (LN) metastasis, distal lymph node metastasis, periductal fat invasion, and adjacent organ invasion. Recorded observations included LN metastasis – defined as an LN with a short axis diameter of at least 1 cm and heterogeneous attenuation or signal intensity on imaging, periductal fat invasion – defined as infiltration of tumor into an adjacent fat plane, and adjacent organ invasion – defined as infiltration of the tumor into a periductal fat plane and adjacent organ.

### 2.3 CT protocol

Thirty-one patients underwent CT imaging using one of two spiral CT scan machines: a 128 spiral CT scanner (Brilliance iCT SP 128 slice, Philips Medical Systems, Netherlands) and a 256-slice spiral dual-source dual-energy CT scanner (Somatom Definition Flash 256 slice, Siemens Medical Solutions, Erlangen, Germany; Table 1). The abdominal CT protocol included a pre-contrast scan and portal venous phase scan (70–80 s after contrast injection). The contrast injection rate was about 5 ml/s (2 ml/kg; not more than 120 ml). Another 13 cases underwent abdominal CT imaging at outside institutions with comparable protocols. Pre-contrast, portal venous-phase, and equilibrium phase images were evaluated using a PACS.

**Table 1:** CT protocols for 128 spiral CT and 256 slice dual-source dual-energy CT scanners.

CT protocol	128 spiral CT scanner	256 dual-source dual-energy CT scanner
Slice thickness	2-mm	2-mm
Kilovoltage	120	120
Tube current	314	156
A pitch	1.2	1.2

### 2.4 MRI protocol

There were 26 cases in which MRI was performed using a 1.5-T MRI system (Magnetom Aera, Siemens Medical Solutions, Erlangen, Germany) and a sixteen-channel body phased-array coil. In another five cases, a 3-T MRI system (Achieva 3.0T TX, Philips Healthcare, Netherlands) and SENSE-XL-Torso coil were used. Another two patients underwent abdominal MRI imaging at outside institutions. Pre contrast, portal venous-phase, and equilibrium phase images were evaluated using a PACS. The MRI protocols of the 1.5-T and 3-T MRI systems are summarized in Table 2.

**Table 2:** MRI-sequence protocols for 1.5-T and 3-T machines.

Sequence	TR 1.5/3 T	TE 1.5/3T	FOV 1.5/3T	Matrix 1.5/3T	Slice thickness 1.5/3T
In-phase T1W	116/134.75	4.84/2.3	400 × 370/320 × 320	320 × 210/308/224	6/6-mm
Opposed phase T1W	116/134.75	2.38/1.15	400 × 370/320 × 320	320 × 210/308/224	6/6-mm
T2W	1200/1015.24	94/70	400 × 370/320 × 320	320 × 208/268 × 268	6/6-mm
Heavily T2W	1200/1304.11	181/180	400 × 370/320 × 320	320 × 208/268 × 268	6/6-mm
DWI with B values 0, 150, and 800 s/mm^2^	7300/2019.68	71/65.35	367 × 340/320 × 320	152 × 154/268 × 268	6/6-mm
3D MRCP	2913/2500	800/70	260 × 260/360 × 360	236 × 237/448 × 412	1/1-mm
Thick-Slab MRCP	4527/4500	750/746	300 × 300/300x/300	320 × 256/384 × 269	5/5-mm
T1W fat saturation, arterial, portovenous, delayed 3,5 min	4.58/3.05	2.16/1.43	400 × 370/320 × 320	320 × 210/212 × 169	3.3/2-mm

### 2.5 Follow-up and recurrence

Post-curative resection follow-up with clinical and radiographic monitoring was performed every 1–6 months by a multidisciplinary team.

Recurrence was determined by documentation of progression on serial CT or MRI cross-sectional imaging with or without elevated carcinogen antigen 19-9 levels. Recurrence was defined as the observation of any new lesion on follow-up imaging with a high degree of confidence. Recurrence patterns were classified as either 1) local, including new soft tissue lesions at the hepatic resection margin, distal bile duct remnant, hepaticojejunostomy, hepatic hilum region, or regional LN metastasis, or 2) distal such as intrahepatic metastasis, distant LN metastasis (including celiac, periaortic, and pericaval LN), or distant organ metastasis such as lung, pleura, brain, bone, adrenal gland, or peritoneal cavity.

Time to recurrence was defined from the time of surgery to the time of first evidence of recurrence on imaging. Recurrence-free survival (RFS) was defined from the time of surgery to the time of first recurrence as evident on imaging or time to death.

Overall survival status was determined from national registries from the date of surgery to the date of death or date of data retrieval. For patients who were alive at the time of data collection without evidence of recurrence on follow-up imaging, data from the last follow-up were documented.

### 2.6 Statistical analysis

Demographic data were analyzed using frequency and percentage for categorical variables and mean and standard deviation (SD) for continuous variables.

All variables in the univariate analysis were included in a Cox proportional hazard model to identify independent significant prognostic factors. Backward selection was used with a 0.1 cutoff for inclusion into the model.

Time to recurrence and RFS were estimated using the Kaplan-Meier method. Univariable analyses were conducted using Kaplan-Meier estimates of survival probabilities and the log-rank test for comparisons. Variables with a p-value less than 0.05 were entered into a Cox proportional hazards regression model for multivariable analyses for both time to recurrence and RFS.

All statistical analyses were performed using STATA (version 10.1. Stata Corp LP, 4905, Lakeway Drive College Station, Texas, USA).

## 3 Results

### 3.1 Patient characteristics and imaging features

During the study period, there were 77 patients with pathologically proven PCCA (28 women and 49 men; age range 41–81 years, mean 60.65 ± 7.66) for whom postoperative follow-up imaging was available. Fifty (65 %) patients experienced recurrence, with 37(48.1 %) patients having already died at the time of data collection. The remaining 27(35.06 %) experienced no recurrence after surgical treatment. Patient characteristics, including demographic data and preoperative imaging features, are shown in Table 3. Adjacent organ invasion, regional LN metastasis, and surgical margin differed significantly between those with recurrent and non-recurrent PCCA.

**Table 3:** Comparison of patient characteristics and imaging variables between recurrent and non-recurrent PCCA after surgical resection.

Variable	Total (n = 77)(%)	Non-recurrence 27 (35.06)	Recurrence 50 (65)	p value
**Sex**				0.162
Female	28 (36.36)	7 (25.93)	21 (42)	
Male	49 (63.64)	20 (74.07)	29 (58)	
**Age (years)**				
Mean (SD)	60.65 (7.66)	63.04 (4.43)	59.36 (8.7)	0.044
Median (min-max)	61 (41–81)	64 (53–70)	58.5 (41–81)	
Median (IQR)	61 (57–65)	64 (59–67)	58.5 (54–64)	
**Bismuth classification**				0.476
1	3 (3.9)	1 (3.7)	2 (4)	
2	1 (1.3)	0 (0)	1 (2)	
3a	39 (50.65)	11 (40.74)	28 (56)	
3b	27 (35.06)	13 (48.15)	14 (28)	
4	7 (9.09)	2 (7.41)	5 (10)	
**Tumor type**				
Periductal	53 (68.83)	18 (66.67)	35 (70)	0.763
Combined	18 (23.38)	5 (18.52)	13 (26)	0.459
Intraductal	7 (9.09)	5 (18.52)	2 (4)	0.048
Mass	0 (0)	0 (0)	0 (0)	NA
**Chronic liver disease**				0.351
Absent	76 (98.7)	26 (96.3)	50 (100)	
Present	1 (1.3)	1 (3.7)	0 (0)	
**Size**				0.706
Non-measurable	55 (71.43)	20 (74.07)	35 (70)	
Measurable	22 (28.57)	7 (25.93)	15 (30)	
**Lobar atrophy**				0.385
No	41 (53.25)	16 (59.26)	25 (50)	
Right	20 (25.97)	5 (18.52)	15 (30)	
Left	15 (19.48)	5 (18.52)	10 (20)	
Both	1 (1.3)	1 (3.7)	0 (0)	
**Vascular invasion**				
**Portal vein invasion**				0.685
No	28 (36.36)	9 (33.33)	19 (38)	
Yes	49 (63.64)	18 (66.67)	31 (62)	
**Hepatic vein invasion**				0.530
No	68 (88.31)	23 (85.19)	45 (90)	
Yes	9 (11.69)	4 (14.81)	5 (10)	
**Hepatic artery**				>0.999
No	76 (98.7)	27 (100)	49 (98)	
Yes	1 (1.3)	0 (0)	1 (2)	
LN				
**Regional LN metastasis**				0.050
No	55 (71.43)	23 (85.19)	32 (64)	
Yes	22 (28.57)	4 (14.81)	18 (36)	
**Distant LN metastasis**				0.173
No	62 (80.52)	24 (88.89)	38 (76)	
Yes	15 (19.48)	3 (11.11)	12 (24)	
**Adjacent organ invasion**				0.049
No	62 (80.52)	25 (92.59)	37 (74)	
Yes	15 (19.48)	2 (7.41)	13 (26)	
**Surgical procedure**				0.087
Right hepatectomy 0	46 (59.74)	12 (44.44)	34 (68)	
Extended right hepatectomy	3 (3.9)	1 (3.7)	2 (4)	
Extended right hepatectomy with caudate lobe resection	1 (1.3)	0 (0)	1 (2)	
Left hepatectomy	23 (29.87)	14 (51.85)	9 (18)	
Extended left hepatectomy	2 (2.6)	0 (0)	2 (4)	
Hilar resection with enterobiliary anastomosis	1 (1.3)	0 (0)	1 (2)	
Segmentectomy	1 (1.3)	0 (0)	1 (2)	
**Surgical margin**				0.003
R0	39 (50.65)	20 (74.07)	19 (38)	
R1	38 (49.35)	7 (25.93)	31 (62)	
R2	0 (0)	0 (0)	0 (0)	

*MF* mass forming*, ID* intraductal*, PD* periductal*, PV* portal vein, *HV* hepatic vein, *HA* hepatic artery, *LN* lymph node, *R0* negative resection margin, *R1* micro positive surgical margin, *R2* macro positive surgical resection margin.

### 3.2 Recurrence-free survival (RFS) analysis

There were four variables significantly associated with recurrence of PCCA after curative resection according to univariate analysis: regional LN metastasis, adjacent organ invasion, surgical procedure, and surgical margin. However, after subsequent multivariable analysis excluding confounding factors only regional LN metastasis, adjacent organ invasion, and surgical margin were independent prognostic factors associated with recurrent disease after post-surgical resection of PCCA. Patients with regional LN metastasis and adjacent organ invasion had a 2.01 and 2.05 times greater rate of recurrence than those without (p = 0.023, 0.028). Those with an R1 positive margin had a 2.15 times greater rate of recurrence than those with an R0 margin (p = 0.010; Table 4.).

**Table 4:** Univariable and multivariable analyses for prognostic factors associated with RFS in recurrent PCCA.

Prognostic factor	Univariable analysis			Multivariable analysis		
	HR	95%CI	p Value	HR	95%CI	p Value
**Size**			0.197			
Non-measurable	1					
Measurable	1.49	0.81–2.75				
**PV invasion**			0.627			
No	1					
Yes	0.87	0.49–1.54				
**HV invasion**			0.500			
No	1					
Yes	0.73	0.29–1.84				
**Regional LN metastasis**			0.005			0.023
No	1			1		
Yes	2.34	1.29–4.22		2.01	1.10–3.67	
**Distant LN metastasis**			0.275			
No	1					
Yes	1.44	0.75–2.78				
**Periductal fat invasion**			0.059			
No	1					
Yes	1.91	0.97–3.74				
**Adjacent organ invasion**			0.015			0.028
No	1			1		
Yes	2.20	1.17–4.17		2.05	1.07–3.89	
**Intrahepatic metastasis**			0.532			
No	1					
Yes	1.34	0.53–3.4				
**Distant metastasis**			0.987			
No	1					
Yes	0.99	0.31–3.20				
**Surgical procedure**			0.033			
Right hepatectomy	1					
Extended right hepatectomy	1.45	0.35–6.16				
Extended right hepatectomy with caudate lobe resection	3.49	0.46–26.57				
Left hepatectomy	0.41	0.2–0.86				
Extended left hepatectomy	3.2	0.75–13.68				
Hilar resection with enterobiliary anastomosis	4.65	0.6–35.93				
Segmentectomy	3.74	0.49–28.57				
**Surgical margin**			0.003			0.010
R0 negative both margins	1			1		
R1 Gross neg - micro positive margin	2.4	1.35–4.25		2.15	1.20–3.86	

**Fig. 1:**
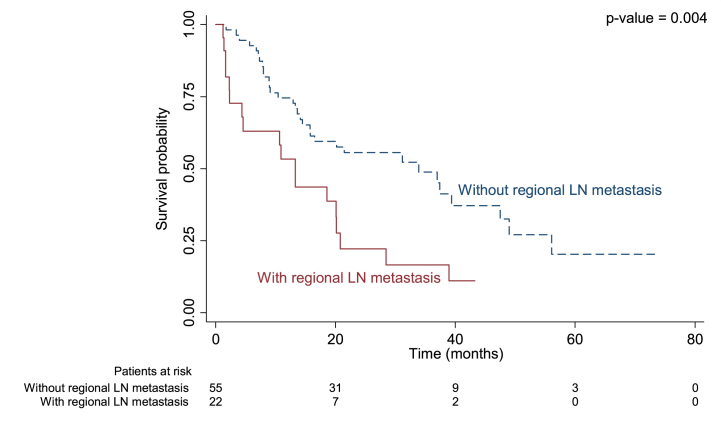
Kaplan-Meier curve for median recurrence-free survival in perihilar cholangiocarcinoma with and without regional LN metastasis.

**Fig. 2:**
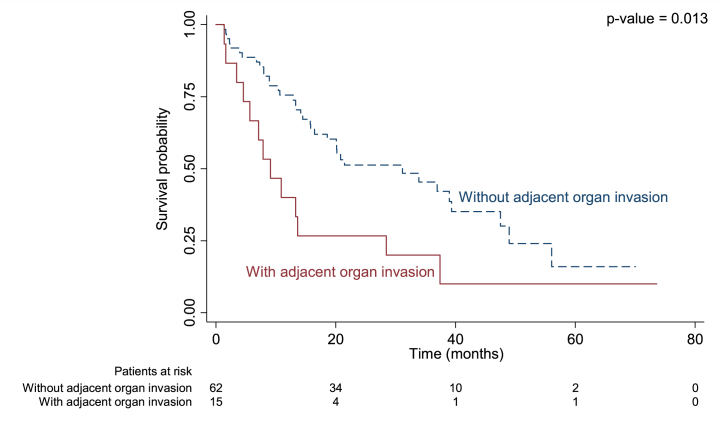
Kaplan-Meier curve for median recurrence-free survival in perihilar cholangiocarcinoma with and without adjacent organ invasion.

**Fig. 3:**
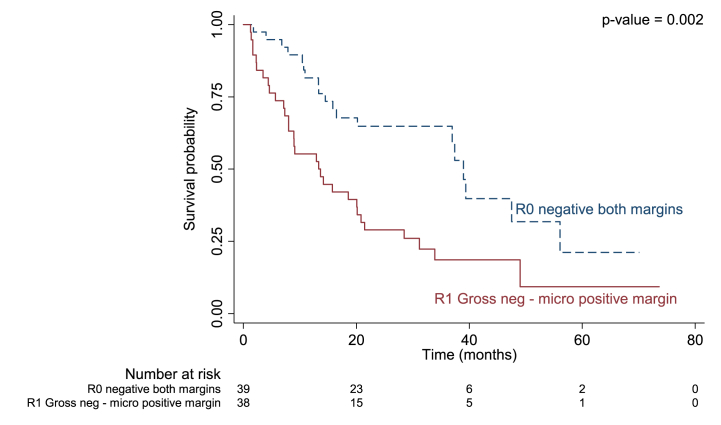
Kaplan-Meier curve for median recurrence-free survival in perihilar cholangiocarcinoma with R0 and R1 post-surgical margin.

Median duration of recurrence-free survival was 13.3 months in patients with regional LN metastasis versus 33.9 months in those without (p = 0.004; Fig. 1), 9.1 months in those with adjacent organ invasion versus 31.2 months (p = 0.013; Fig. 2), and 13.7 months in those with an R1 positive margin compared to 38.9 months in those with R0 (p = 0.002; Fig. 3).

### 3.3 Comparison of imaging features and overall survival analysis (OS)

**Fig. 4:**
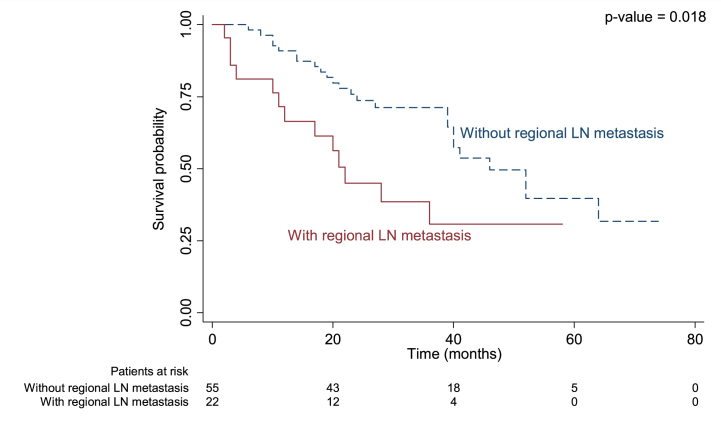
Kaplan-Meier curve for overall survival (OS) in perihilar cholangiocarcinoma with and without regional LN metastasis.

**Fig. 5:**
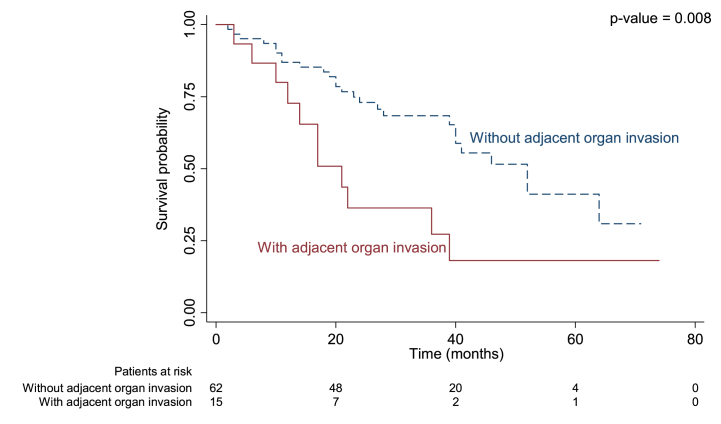
Kaplan-Meier curve for overall survival (OS) in perihilar cholangiocarcinoma with and without adjacent organ invasion.

**Fig. 6:**
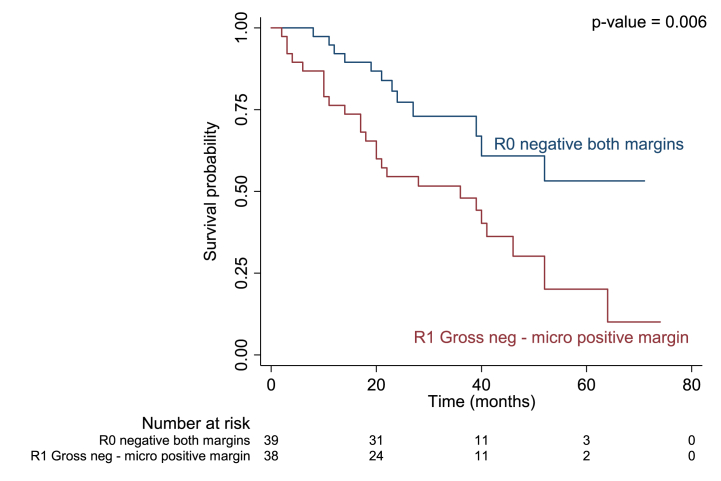
Kaplan-Meier curve for overall survival (OS) in perihilar cholangiocarcinoma with R0 and R1 post-surgical resection margin.

There were four variables associated with OS according to univariate analysis: regional LN metastasis, periductal fat invasion, adjacent organ invasion, and intrahepatic metastasis (p = 0.022, p = 0.020, p = 0.011, p = 0.026, respectively; Table 5.). However, only regional LN metastasis, adjacent organ invasion, and post-surgical margin remained significant after multivariable analysis. The mortality rate was 2.08 times higher in patients with regional LN metastasis, 2.39 times higher in those with adjacent organ invasion, and 2.43 times higher in those with R1 resection margin (p = 0.040, p = 0.018, p = 0.013, respectively; Table 5.) Median OS was 22 months in those with regional LN metastasis compared to 46 months in those without (p < 0.018, Fig. 4), 21 months in those with adjacent organ invasion versus 52 months (p = 0.008, Fig. 5), and 36 months in those with R1 resection margin compared to 51.56 in those with R0 (p = 0.006, Fig. 6).

**Table 5:** Univariable and multivariable analyses for prognostic factors associated with OS in recurrent PCCA.

Prognostic factor	Univariable analysis			Multivariable analysis		
	HR	95%CI	p Value	HR	95%CI	p Value
**Size**			0.076			
Non-measurable	1					
Measurable	1.85	0.94–3.67				
**PV invasion**			0.459			
No	1					
Yes	1.30	0.65–2.59				
**HV invasion**			0.623			
No	1					
Yes	1.27	0.49–3.27				
**Regional LN metastasis**			0.022			0.040
No	1			1		
Yes	2.23	1.12–4.42		2.08	1.03–4.16	
**Distant LN metastasis**			0.400			
No	1					
Yes	1.41	0.64–3.11				
**Periductal fat invasion**			0.020			
No	1					
Yes	2.38	1.15–4.94				
**Adjacent organ invasion**			0.011			0.18
No	1			1		
Yes	2.53	1.24–5.15		2.39	1.16–4.9	
**Intrahepatic metastasis**			0.026			
No	1					
Yes	2.73	1.13–6.61				
**Distant metastasis**			0.542			
No	1					
Yes	1.45	0.44–4.77				
**Surgical procedure**			0.085			
Right hepatectomy	1					
Extended right hepatectomy	2.16	0.5–9.38				
Extended right hepatectomy with caudate lobe resection	3.29	0.43–25.04				
Left hepatectomy	0.46	0.19–1.12				
Extended left hepatectomy	7.79	1.74–34.98				
Hilar resection with enterobiliary anastomosis	1.14	0.16–8.52				
Segmentectomy	1.60	0.22–11.93				
**Surgical margin**			0.008			0.013
R0 negative both margins	1			1		
R1 Gross neg - micro positive margin	2.55	1.28–5.1		2.43	1.21–4.87	
R2 Gross + micro positive margin	–	–

### 3.4 Recurrence patterns and recurrence-free survival

**Fig. 7:**
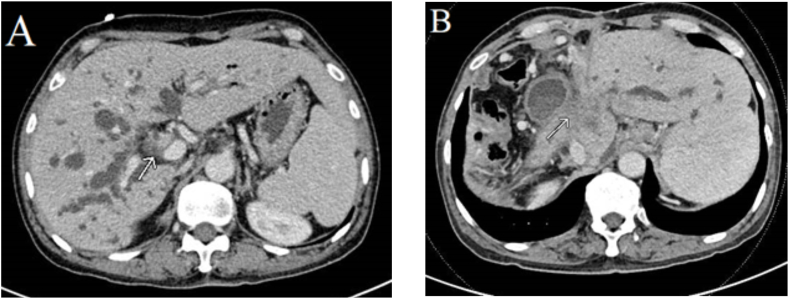
A 60-year-old man with perihilar cholangiocarcinoma with periductal fat invasion (white arrow; Bismuth-Corellete 3A) on pre-operative imaging (Fig. 7A). Post-curative resection follow-up CT at 13 months shows infiltration of a recurrent tumor at the surgical bed (white arrow; Fig. 7B).

**Fig. 8:**
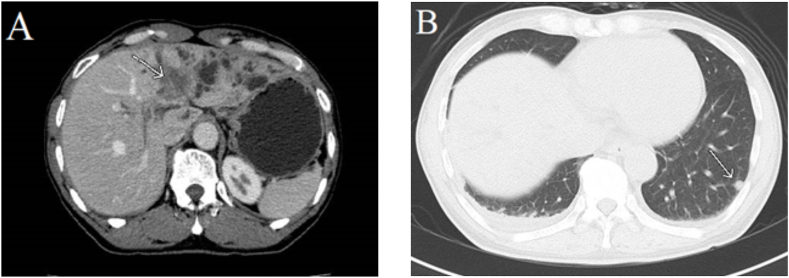
A 59-year-old man with perihilar cholangiocarcinoma. Portovenous phase CT shows an irregular poor enhancing mass at the hilar (white arrow) invading the left portal vein and causing intrahepatic duct dilation and atrophic changes in the left lobe of the liver (Fig. 8A). Post-curative resection follow-up chest CT at 3 months shows evidence of pulmonary metastasis at the lateral segment of the left lower lobe (white arrow; Fig. 8B).

Local recurrence was more common than distant organ metastasis after surgical resection of PCCA (64 % versus 60 %), and the incidence of surgical bed recurrence was higher than that of regional LN metastasis (58 % versus 10 %)**.** Thirty patients (60 %) experienced distant organ metastasis, of which peritoneal seeding (30 %) was the most common, followed by lung metastasis (22 %), liver metastasis (16 %), distal LN metastasis (6 %), and pleural metastasis (2 %; Table 6, Fig. 7, Fig. 8).

**Table 6:** Incidence of recurrence patterns of PCCA post curative resection.

Recurrent pattern	N (%)
**Local recurrence**	32 (64)
Surgical bed recurrence	29 (58)
Regional LN metastasis	5 (10)
**Distant metastasis**	30 (60)
Peritoneal seeding	15 (30)
Lung metastasis	11 (22)
Liver metastasis	8 (16)
Distant LN metastasis	3 (6)
Pleural metastasis	1 (2)

## 4 Discussion

While surgical excision is deemed the optimal curative approach for perihilar cholangiocarcinoma (PCCA), the long-term outlook remains poor, even with complete tumor removal (R0 resection). Contributing to this grim prognosis are factors like lymph node (LN) metastasis, tumor grade, R1 resection, vascular involvement, larger tumor size, compromised patient health, and elevated CA19-9 levels [30,32,33,35]. Crucial to preoperative planning, imaging offers deep insights into prognosis, with multimodal multiparametric imaging particularly effective in providing a thorough understanding of tumor biology and stage, especially in predicting adverse outcomes. This imaging approach aids in prognosis prediction and is instrumental in choosing appropriate treatment strategies [49,50]. Our study focused on identifying pre-surgery radiographic characteristics linked with postoperative recurrence and examining the patterns of recurrence post-surgery.

Our study observed a 65 % recurrence rate post-PCCA surgery, consistent with previous reports indicating rates between 49 % and 66 % [30,33,35,[51], [52], [53], [54], [55]]. Groot Koerkamp et al. [30] highlighted R1 resection margins and LN metastasis as key factors for increased recurrence (76 %) over eight years post-surgery and a median relapse-free survival (RFS) of 26 months. Limitations in record-keeping and patient follow-up in our study might have led to an underestimation of these recurrence rates. We identified regional lymph node metastasis, adjacent organ invasion, and the presence of R1 surgical margins as key prognostic factors for recurrence, significantly correlating with shorter overall survival (p = 0.040, p = 0.018, p = 0.013, respectively).

Our results showed patients with regional lymph node involvement exhibited a 2.21-fold higher recurrence rate (p = 0.023) and a 2.10-fold increase in mortality compared to those without such involvement. The median RFS for patients with LN metastasis was 13.3 months, versus 33.9 months for others (p = 0.004), and the median overall survival (OS) was 22 months compared to 46 months (p = 0.018). This is in keeping with previous publications which have also shown that the presence of lymph node (LN) metastasis is one of the most significant factors associated with an unfavorable prognosis for PCCA [21,30,31,33,35,[37], [38], [39],^56^,^57^]. Our study showed a shorter RFS compared to the 26 months reported by Groot Koerkamp et al. [30]. In their study, Groot Koerkamp et al. [30] identified resection margin, LN status, and tumor differentiation as independent factors that were associated with this outcome. The variation in their results may be attributed to the preoperative administration of low-dose radiotherapy, alongside disparities in methodology and protocols. Kobayashi and colleagues [58] found an 80 % recurrence rate after 3 years, increasing to 100 % with longer follow-up. Aoba et al. [59] noted LN metastasis in 45.6 % of PCCA patients as a factor for shorter survival, whereas our study found LN metastasis in 36 % of recurrent cases and 14.81 % in non-recurrent cases (P = 0.05), possibly due to our smaller sample size. Buettner et al. [39] reported improved 5-year OS rates with lymph node resection in PCCA patients.

Advanced imaging techniques can accurately assess and categorize LN metastasis before surgery, influencing decisions about tumor resectability. This capability is affirmed by several studies [49,50], although Promsorn et al. [60] found no significant difference in the apparent diffusion coefficient (ADC) between benign and metastatic LNs in cholangiocarcinoma patients. When imaging indicates possible distant or regional LN metastasis, especially in inoperable patients, a lymph node biopsy is recommended. This preoperative imaging data is pivotal in identifying patients who may benefit from surgery or those for whom it may prolong OS while improving quality of life.

Our study found adjacent organ invasion to be another significant prognostic factor related to recurrence. We found that 26 % of patients with recurrent PCCA had adjacent organ invasion (diaphragm, adrenal gland, gall bladder, adrenal gland, and peritoneum) compared to 7.41 % of those without (p = 0.49). To the best of our knowledge, no other study has been published showing an association between adjacent organ invasion on pre-operative imaging and PCCA prognosis. The 7th and 8th American Joint Committee on Cancer/Union for International Cancer Control (AJCC/UICC) defined T2a staging as tumor invasion to the surrounding adipose tissue beyond the wall of the bile duct and T2b staging as tumor invasion to the adjacent hepatic parenchyma. However, they did not address these criteria in PCCA, nor does Memorial Sloan-Kettering Cancer Center (MSKCC) staging [26]. Our study found that PCCA patients with adjacent organ invasion had a median of 9.1 months RFS compared to 31.2 months in those without (p = 0.013) as well as a shorter survival rate (21 versus 52 months; p = 0.008). Recurrent patients with adjacent organ invasion were also 2.39 times more likely to die than those without according to multivariate analysis (P = 0.018). As adjacent organ invasion was a significant prognostic factor related to recurrence, RFS, and OS, it should be included in tumor staging systems for better prognostic evaluation and to further optimize treatment options.

Finally, we also found surgical margin to be a significant prognostic factor related to recurrent PCCA. Of those who had recurrence after surgical resection 62% had an R1 resection margin versus 25.93 % in those who did not (p = 0.003). Patients with an R1 resection margin were 2.15 times more likely to experience recurrence and 2.43 times more likely to die compared to those with R0 (p = 0.010 and p = 0.013, respectively). In addition, RFS was 13.7 months and OS was 36 months in those with R1 positive margins versus 38.9 months and 51.56 months, in those with R0 margin (p = 0.002, p = 0.006, respectively). These results are comparable to those of Groot Koerkamp et al. [30], who found that patients with a positive or narrow resection margin were 2.25 times more likely to experience recurrence. However, they also found a longer time to recurrence of 31 months, which may have been due to those patients undergoing radiation treatment prior to surgery. Our study also found that 38 % of patients who experienced recurrence had an R0 resection margin compared to 74 % of non-recurrent cases. Another previous study also reported recurrence among PCCA patients with R0 resection margins and tumor at least 5 cm, LN metastasis, and/or venous invasion [33,35]. Adjuvant treatment options, such as radiation, should thus be considered in this type of malignant tumor, even in patients with an R0 resection margin.

The other variables examined in our study, including demographic data, Bismuth classification, morphologic type, underlying liver disease, lobar atrophy, vascular invasion, and distant LN metastasis, were not associated with disease recurrence. This differs from other reports that have found tumor differentiation, vascular invasion, large tumor, poor performance status, and elevated CA19-9 to be significantly related to PCCA recurrence [30,32,33,35].

We also evaluated postoperative recurrence patterns on follow-up imaging and found a 64 % rate of local recurrence (58 % surgical bed recurrence and 10 % regional LN metastasis) and a 60 % incidence of distant metastasis (30 % peritoneal seeding, 22 % lung metastasis, 16 % liver metastasis, 6 % distant LN metastasis and 2 % pleural metastasis). By contrast, Groot Koerkamp et al. [30] reported a 26 % local recurrence rate (19 % liver hilum, 13 % hepatojejunostoma, 13 % liver section margin, and 3 % distal bile duct remnant) and a distant metastasis rate of 40 % (24 % retroperitoneal LN, 23 % intrahepatic metastasis, 21 % peritoneum, 14 % lung or mediastinum, 5 % abdominal wall/incision, 2 % bone, and 1 % each of skin, adrenal gland, axillary or neck LN, and spleen).

Our study had several limitations. The first was our smaller sample size compared to previously published studies, which likely led to some variables being non-significant. Second, the post-curative surgical resection period was incomplete due to loss to follow-up. Finally, there were a variety of imaging modalities employed in this study, leading to heterogeneous imaging data. Further prospective studies with better cohort control might provide better results.

## 5 Conclusions

We found that pre-surgical imaging of regional LN metastasis, adjacent periductal fat or adjacent organ invasion, and R1 resection margin were associated with recurrent PCCA and shorter OS. Therefore, periductal fat or adjacent organ invasion should be addressed in the PCCA staging system to optimize the accuracy of tumor staging, prognosis prediction, and determination of treatment options.

## Ethics approval and consent to participate

Ethics approval was provided by the Ethics Committee of the Faculty of Medicine, Khon Kaen University, as instituted by the Helsinki Declaration, and this study was retrospective study fort this type of study formal consent is not required. The reference number of ethical approval is HE611023.

## Consent for publication

All images in this manuscript contain no individual personal data.

## Availability of data and material

The datasets used and/or analyzed during the current study are available from the corresponding author on reasonable request.

## Competing interests

The authors declare that they have no competing interests.

## Funding

This study was supported by Research and Graduate Studies, 10.13039/501100004071Khon Kaen University, Thailand.

## Data availability statement

The data that support the findings of this study are available from the corresponding author, [JP] upon reasonable request.

## CRediT authorship contribution statement

**Julaluck Promsorn:** Writing – review & editing, Writing – original draft, Visualization, Validation, Supervision, Project administration, Methodology, Investigation, Funding acquisition, Formal analysis, Data curation, Conceptualization. **Panjaporn Naknan:** Writing – original draft, Validation, Software, Data curation. **Aumkhae Sookprasert:** Visualization, Investigation. **Kosin Wirasorn:** Visualization, Investigation. **Jarin Chindaprasirt:** Visualization, Investigation. **Attapol Titapun:** Visualization, Investigation. **Piyapharom Intarawichian:** Visualization, Investigation. **Mukesh Harisinghani:** Writing – review & editing, Supervision.

## Declaration of competing interest

The authors declare that they have no known competing financial interests or personal relationships that could have appeared to influence the work reported in this paper.

## Abbreviations

American Joint Commission on Cancer: AJCC
Perihilar cholangiocarcinoma: PCCA
Lymph node: LN
Overall survival: OS
Cholangiocarcinoma: CCA
American Joint Commission on Cancer: AJCC
Health object: HO
Computed Tomography: CT
Magnetic Resonance Imaging: MRI
Standard deviation: SD
Portal vein: PV
Hepatic vein: HV
